# Effects of Troxerutin on Oxidative Stress, Inflammation and Galectin- 3 Expression in Intracerebroventricular Kainic Acid-Induced Neurotoxicity

**DOI:** 10.1007/s10753-025-02301-9

**Published:** 2025-04-16

**Authors:** Mehmet Demir, Hulya Elbe, Dilan Cetinavci, Ercan Saruhan

**Affiliations:** 1 Department of Physiology, Faculty of Medicine, Karabuk University, Karabuk, Turkey; 2 Department of Histology and Embryology, Faculty of Medicine, Mugla Sıtkı Kocman University, Mugla, Turkey; 3 Department of Histology and Embryology, Mugla Training and Research Hospital, Mugla, Turkey; 4 Department of Medical Biochemistry, Faculty of Medicine, Mugla Sıtkı Kocman University, Mugla, Turkey

**Keywords:** Troxerutin, Intracerebroventricular, Kainic acid, Oxidative stress, Inflammation, Galectin- 3

## Abstract

Excitotoxicity caused by excessive concentration of the excitatory neurotransmitter glutamate causes neuronal cell death and promotes neurodegenerative disorders. The neuroexcitant neurotoxin kainic acid (KA) induces excitotoxicity, leading to neuronal death via oxidative stress and inflammation, and its experimental use is widespread. This study was designed to determine the protective effect of Troxerutin (TXR) and its relationship with Galectin-3 (Gal-3) in experimental excitotoxicity with neuroinflammation and oxidative stress. Fifty male Wistar rats were divided into five groups (*n* = 10): Control group rats received intraperitoneal (ip) normal saline for 6 days. Sham group rats received a single dose of intracerebroventricular (icv) normal saline on the first day. KA group rats were treated with a single dose of KA; icv-0.5 μg/μl). TXR group rats treated with TXR for 6 days: ip-100 mg/kg) and KA + TXR group rats treated with KA (single dose) and TXR (6 days). It was observed that malondialdehyde (MDA) and interleukin-1β (IL-1β) levels increased and reduced glutathione (GSH) levels decreased in the cerebral cortex of rats with KA neurotoxicity. TXR treatment caused a significant improvement in MDA and GSH levels and a significant decrease in IL-1β levels in rats with the excitotoxicity model. Gal-3 expressions in the hippocampus and cerebellum increased in KA-treated rats, whereas TXR treatment decreased Gal-3 expressions. In addition, histopathological changes caused by KA administration showed improvement in TXR-treated groups. In conclusion, the findings showed that TXR treatment attenuated KA-induced neurotoxicity by reducing oxidative tissue damage, inflammatory response and Gal-3 expression.

**Graphical Abstract:**

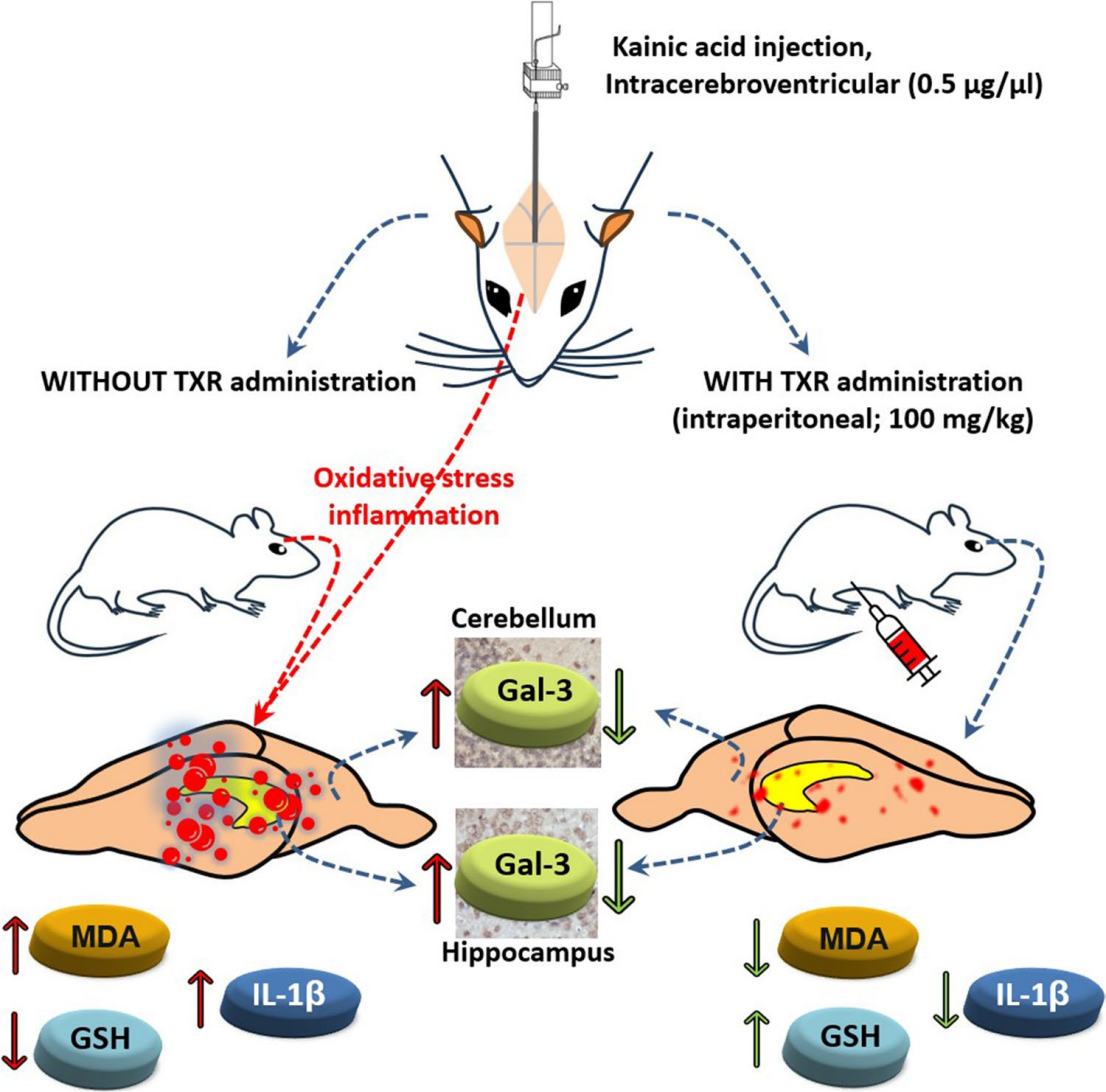

## Introduction

Glutamate, the brain's main excitatory neurotransmitter, is released into the synaptic cleft under physiological conditions, taken into the cells by glutamate transporters in the membranes of postsynaptic neurons and cleared from the synaptic cleft [1]. Glutamate binds to two receptor types: ionotropic glutamate and metabotropic glutamate receptors [2]. Ionotropic glutamate receptors allow ions to pass through the postsynaptic membrane, whereas metabotropic glutamate receptors activate signalling cascades through second messengers [3]. Ionotropic glutamate receptors are divided into three subtypes: N-methyl-D-aspartic acid (NMDA), α-amino- 3-hydroxy- 5-methylisoxazole- 4-propionate (AMPA) and kainic acid (KA) [4].

Excitotoxicity is defined as neuronal damage caused by overexcitation of ionotropic glutamate receptors and excessive glutamate release into the synaptic cleft [5–7]. Excitotoxicity has been reported to play an important role in different brain pathologies involving neuroinflammation and oxidative stress, such as cerebral strokes, epilepsy and neurodegenerative disorders [3, 8]. This is manifested by beading and fragmentation of neurites, especially when exposed to excitotoxins such as the AMPA receptor agonist KA [9]. KA exhibits potent neuroexcitant and neurotoxin properties [10, 11], increasing synaptic activity and causing seizures, neurodegeneration and remodelling [12]. The experimental application of KA, either systemically or intracerebrally, is prevalent and favoured for inducing lesions in neuronal cell bodies inside the central nervous system; even the systemic treatment of KA in rats is a recognised model for temporal lobe epilepsy [13]. KA administration results in spontaneous recurring seizures and a persistent latent phase culminating in epilepsy [14].

Many studies have suggested that KA induces excitotoxicity, leading to neuronal death through oxidative stress and inflammation [15–18]. Systemic or intracerebroventricular (icv) injection of KA causes neurodegeneration in many regions of the brain (piriform cortex, amygdala, cortical and limbic) [19–21] in rodents, especially in the hippocampal subregions of CA1 and CA3 and the hilus of the dentate gyrus [18]. It is also suggested that KA injections directly into the cerebellum selectively destroy stellate, basket and Golgi II cells, especially Purkinje cells [22].

Galectin 3 (Gal- 3), a glycan-binding protein, is increased in some neurodegenerative diseases with neuronal loss and brain injury and is thus suggested to regulate astrocyte proliferation, microglial activation and inflammation in injury [23–25]. Gal- 3, a regulator of microglial activation [26], was detected in the cerebral cortex, thalamus, corpus callosum and hippocampus 72 h after perinatal rat hypoxic-ischemic brain injury [27]. Furthermore, Gal- 3 is widely distributed in neurons and glial cells in various brain regions, including the brainstem and cerebellum [28]. Gal- 3 has been shown to be involved in processes such as inflammation, tissue fibrosis, angiogenesis, apoptosis, cell differentiation [29–31]. It is suggested that inhibition of the activity of this protein leads to a significant decrease in the inflammatory response [32]. Therefore, Gal- 3 is considered as a therapeutic target and a potential biomarker [33].

Troxerutin (TXR) is a flavonoid with multiple biological properties and diverse pharmacological activities in vitro and in vivo [34–36]. Water-soluble TXR [37], can be isolated from tea, coffee, cereals, and various fruits and vegetables [35, 38]. TXR has a high potential to cross the blood–brain barrier. [39]. It is easily absorbed by the gastrointestinal system [40]. It has been suggested that TXR shows a cytoprotective effect in different cell types with its free radical scavenging ability [35]. Furthermore, it has been documented that TXR can reduce the infiltration of inflammatory cells and the expression of inflammation-related proteins and proinflammatory cytokines in various tissues [41–43]. In experimental studies conducted by creating some pathologies, TXR has been proven to improve neurological functions and reduce blood–brain barrier permeability and free oxygen radicals [44]. At the same time, it has been shown to significantly regulate cardiac rhythm, arterial pressure and blood sugar levels in some pathologies [45]. Therefore, in recent years, TXR has been seen as a potential agent with various pharmacological and therapeutic activities, including antioxidant, anti-inflammatory, anti-diabetic and anti-tumour [46–48].

All these data suggest that TXR may show protective effects by suppressing the inflammatory response and oxidative stress in the brain caused by icv KA administration. Therefore, the present study was designed to evaluate the possible protective effect of TXR in excitotoxicity involving neuroinflammation and oxidative stress. For this purpose, tissue oxidative damage levels were analysed by examining malondialdehyde (MDA) and reduced glutathione (GSH) in brain tissue. In addition, interleukin- 1β (IL- 1β) and Gal- 3 levels were analysed to determine inflammation in brain tissue. In addition, histopathological examinations were performed to observe cerebellum and hippocampus damage.

## Materials and Methods

### Ethical Approval

The protocol was approved by the Karabuk University Animal Experiments Local Ethics Committee (protocol number 2024/01/01). Rats were purchased from Karabuk University Experimental Medicine Application and Research Centre (DETUM), and the study was conducted in DETUM as specified in the ethics committee protocol.

### Experimental Group and Treatment

Fifty male Wistar rats (225–250 g) were divided into five groups (n = 10): (1) control group (Rats treated with TXR solvent (normal saline) for 6 days) [47]; (2) sham group (Rats treated with a single dose of KA solvent (normal saline)); (3) KA group (Rats treated with a single dose of KA; 0,5 μg/μl) [49, 50]; (4) TXR group (Rats treated with TXR for 6 days) [42]; and (5) KA + TXR group (Rats treated with KA (single dose) and TXR (for 6 days)). The animals received daily intraperitoneal (ip) injections of TXR at a dose of 100 mg/kg, or normal saline for 6 days after icv injection. After the procedures in all these groups (on the 7 th day), rats were sacrificed under anesthesia (ketamine/xylazine (80/8 mg/kg)) [51, 52]. The cerebral cortex, hippocampus and cerebellum tissues were removed, and biochemical and histopathological examinations were performed.

### Intracerebroventricular (icv) Kainic Acid Injection

Lateral ventricular injection was performed using the bregma point as a reference and Paxinos-Watson coordinates (AP = − 0.8, ML = 1.4, DV = 3.3) [53, 54]. KA (0.5 μg/μl) dissolved in normal saline [49, 50] was injected icv (5 μl/kg) via Hamilton microsyringe [54]. The injected needle was not removed for 2 min to prevent back diffusion of the solution [55]. Then, the needle was slowly withdrawn from the brain, and the scalp was sutured.

### Biochemical Analyses

Oxidative damage markers (MDA, GSH) were analysed by colorimetric method, inflammation marker (IL- 1β) by enzyme-linked immunosorbent assay (ELISA) in cerebral cortex tissue. On the day of the study, brain tissue was sunghed and then homogenised 1/10 in cold phosphate buffer (PBS; pH: 7.4; 50 mM) using a homogeniser (IKA T10 Ultra-Turrax 10) at 20,000 rpm. Tissue homogenate was centrifuged at 14000 rpm for 5 min, and ELISA and colourimetric methods performed MDA, GSH and IL- 1β tests. MDA and GSH were analysed using the Colorimetric Assay Kit for rats (Elabscience, USA; Cat. number: E-BC-K025-M and E-BC-K030-M, respectively). IL- 1β concentration was analysed using an IL- 1β ELISA kit for rats (Elabscience, USA; Cat. number: E-EL-R0012).

### Histopathological Evaluation

The hippocampus and cerebellum were fixed in formalin for 48 h and processed into a subset of paraffin blocks. On the same day, sections (five μm thick) from the tissue blocks were taken on slides and stained with Haematoxylin Eosin (H&E) and Cresyl Violet. Subsequently, microscopic examination was conducted utilising stained preparations at X20 and X40 magnification, using a light microscope (Nikon Eclipse 80i and Nikon Image Analysis System).

The number of pyknotic nuclei and hemorrhagic areas for the hippocampus, and pyknotic nuclei and normal Purkinje cells for the cerebellum were counted. For this analysis, 10 different fields were evaluated from each animal (n = 10) section at X40 magnification (100 different fields for each group).

### Immunohistochemical (IHC) Evaluation

The hippocampus and cerebellum sections were obtained through paraffin blocks and then placed on polylysine-coated slides for further processing in a staining process explicitly designed for immunohistochemical applications. After rehydration, tissue sections were placed in citrate buffer (pH 7.6) and incubated in a microwave oven for 20 min. The samples were then cooled at room temperature for 20 min and washed with phosphate-buffered saline (PBS). Tissue sections were incubated in 0.3% H2O2 for 7 min and washed with PBS. Following this, an anti-Gal- 3 antibody (1:80, GTX125897, GeneTex, Inc., North America) was applied to the sections for 60 min, after which the sections were washed with PBS. Subsequently, sections were maintained in biotinylated antipolyvalent for 10 min, followed by an additional 10 min of exposure to streptavidin peroxidase at room temperature. Chromogen + substrate was applied to the slides and then counterstained with Mayer's haematoxylin. Following a thorough wash with tap water, the sections underwent a process of dehydration. Subsequent to this, the standard manufacturer's instructions for performing the antibody procedure were followed. The formation of a brown color indicated the presence of Gal- 3.

#### Evaluation of IHC Staining

Semi-quantitative H-Score values were calculated by counting positively stained cells in 5 randomly selected fields for each group. The staining intensity in the cell was scored as 0, 1, 2, or 3 corresponding to the presence of negative, weak, intermediate, and strong brown staining, respectively. The total number of cells in each field and the number of cells stained at each intensity were counted and the average percentage positive was calculated for each staining intensity of 0, 1, 2, and 3. And the following formula was applied: H-score: (% of cells stained at intensity category 1 × 1) + (% of cells stained at intensity category 2 × 2) + (% of cells stained at intensity category 3 × 3) [56]. Immunostaining coverslips were evaluated under a light microscope (Nikon Eclipse 80i and Nikon Image Analysis System).

### Statistical Analysis

Statistical analysis was performed using SPSS version 27 (SPSS Inc., Chicago, USA). Shapiro–Wilk test was used to evaluate the conformity of the data to normal distribution. ANOVA test was used to evaluate the data that conformed to normal distribution. Bonferroni test and Tamhane’s T2 were used as post-hoc test for data evaluated with ANOVA. Results were expressed as mean ± standard deviation. In the interpretation of statistical data, *p* < 0.05 was considered significant (Table 1).

**Table 1:** Quantitative histopathological findings in the hippocampus and cerebellum among the experimental groups

Groups	Number of pyknotic nuclei in the hippocampus	Number of hemorrhagic areas in the hippocampus	Number of pycnotic cells in the cerebellum	Number of Purkinje cells in the cerebellum
Group 1: Control	0.12 ± 0.33	0.01 ± 0.10	1.11 ± 0.55	7.36 ± 0.75
Group 2: Sham	0.14 ± 0.35	0.06 ± 0.28	1.12 ± 0.54	7.37 ± 0.76
Group 3: KA	2.46 ± 0.52^a^	0.45 ± 0.50^a^	3.64 ± 0.98^a^	4.89 ± 0.78^a^
Group 4: TXR	0.13 ± 0.34	0.01 ± 0.10	1.16 ± 0.60	7.36 ± 0.75
Group 5: KA + TXR	0.19 ± 0.39	0.07 ± 0.26	1.19 ± 0.60	7.34 ± 0.74

Data are expressed as mean ± SE (For this evaluation, 10 different fields were evaluated from each animal (*n* = 10) section at X40 magnification (100 different fields for each group)

^a^KA vs Control, Sham, TXR and KA + TXR *P* < 0.001

## Results

### Biochemical Findings

#### Oxidative Stress

**Fig. 1:**
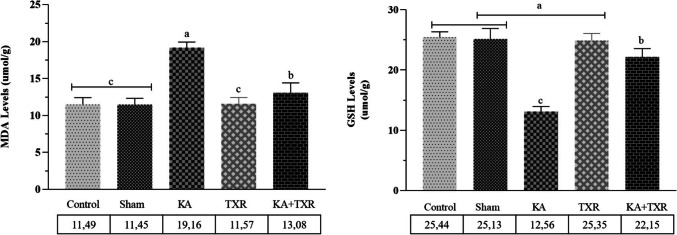
Results of tissue MDA and GSH levels. Data are expressed mean ± SD (*n* = 10). *p* < 0.001 was regarded as significant. Groups marked with different letters were statistically different from each other (*p* < 0.001). ^a^KA vs ^c^Sham and ^a^KA vs ^c^Control P < 0.001, ^a^KA vs ^c^TXR and ^a^KA vs ^b^KA + TXR P < 0.001, ^c^TXR vs ^b^KA + TXR P < 0.001, ^b^KA + TXR vs ^c^Control and ^b^KA + TXR vs ^c^Sham *P* < 0.001 (for MDA). ^c^KA vs ^a^Sham and ^c^KA vs ^a^Control and ^c^KA vs ^a^TXR P < 0.001, ^c^KA vs ^b^KA + TXR P < 0.001, ^b^KA + TXR vs ^a^Control and ^b^KA + TXR vs ^a^Sham and ^b^KA + TXR vs ^a^TXR *P* < 0.001 (for GSH)

In KA-induced excitotoxicity, tissue oxidative damage levels were analysed by examining MDA and GSH in the cerebral cortex. Comparison of the excitotoxic KA group with the control, sham and TXR groups showed a significant increase in brain tissue MDA levels and a significant decrease in GSH levels in the KA group rats (*p* < 0.001). On the other hand, TXR treatment (KA + TXR) in the KA group resulted in a significant improvement (*p* < 0.001) in MDA and GSH levels compared to the excitotoxicity model group (KA group) (Fig. 1).

#### Inflammatory Factor

**Fig. 2:**
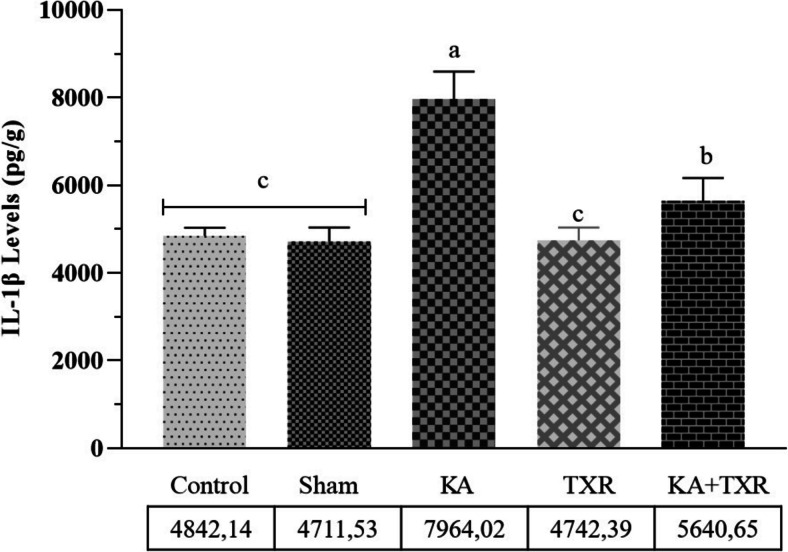
Results of tissue IL- 1β levels. Data are expressed mean ± SD (*n* = 10). *p* < 0.001 was regarded as significant. Groups marked with different letters were statistically different from each other (*p* < 0.001). ^a^KA vs ^c^Sham and ^a^KA vs ^c^Control *P* < 0.001, ^a^KA vs ^c^TXR and ^a^KA vs ^b^KA + TXR *P* < 0.001, ^c^TXR vs ^b^KA + TXR *P* < 0.001, ^b^KA + TXR vs ^c^Control and ^b^KA + TXR vs ^c^Sham *P* < 0.001

We evaluated cerebral inflammation after KA-induced excitotoxicity and the protective potential of TXR by measuring IL- 1β levels in cerebral cortex tissue. As presented in (Fig. 2), our results showed that IL- 1β levels of the model group (KA group) were significantly increased compared to Control, Sham and even TXR groups (*p* < 0.001). In addition, TXR treatment of the model group (KA group) rats (KA + TXR group) caused a statistically significant decrease in cerebral cortex IL- 1β levels compared to the KA administered group (KA group) (*p* < 0.001).

#### Histopathological Findings

**Fig. 3:**
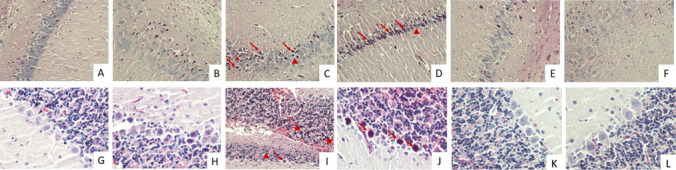
Images of Hematoxylin–Eosin (H&E) staining of hippocampus and cerebellum. Dark eosinophilic cytoplasm (arrow), pyknotic nuclei (arrowhead), and haemorrhage (asteriks) were observed. **A-F**: Hippocampus, **G-L**: Cerebellum. **A**. Control group, H-E, X40. **B**. Sham group, H-E, X40. **C**. KA group, H-E, X40. **D**. KA group, H-E, X20. **E**. TXR group, H-E, X40. **F**. KA + TXR group, H-E, X40. **G**. Control group, H-E, X40. **H**. Sham group, H-E, X40. **I**. KA group, H-E, X20. **J**. KA group, H-E, X40. **K**. TXR group, H-E, X40. **L**. KA + TXR group, H-E, X40

**Fig. 4:**
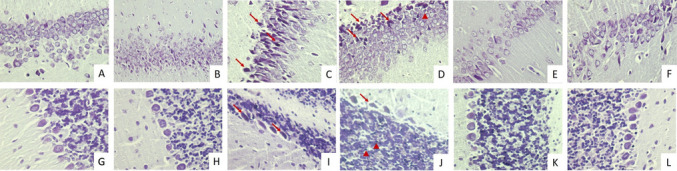
Images of Cresyl Violet (CV) staining of hippocampus and cerebellum. Dark eosinophilic cytoplasm (arrow), and pyknotic nuclei (arrowhead) were observed. **A-F**: Hippocampus, **G-L**: Cerebellum. **A**. Control group, X40. **B**. Sham group, X40. **C**. KA group, X40. **D**. KA group, X40. **E**. TXR group, X40. **F**. KA + TXR group, X40. **G**. Control group, X40. **H**. Sham group, X40. **I**. KA group, X40. **J**. KA group X40. **K**. TXR group, X40. **L**. KA + TXR group, X40

The study focused on the CA1 region of the hippocampus and cerebellum. It was observed that the cells in the hippocampus in the control, sham, and TXR groups were normal density, neuron cells were arranged regularly and the neuron cytoplasm and nucleus showed normal staining properties and typical morphology (Figs. 3A, B, E and 4A, B, E). It was observed that the moleculare, gangliosum, and granulosum layers and white matter of the cerebellum were normal in the control, sham and TXR groups (Figs. 3G, H, K and 4G, H, K).

**Fig. 5:**
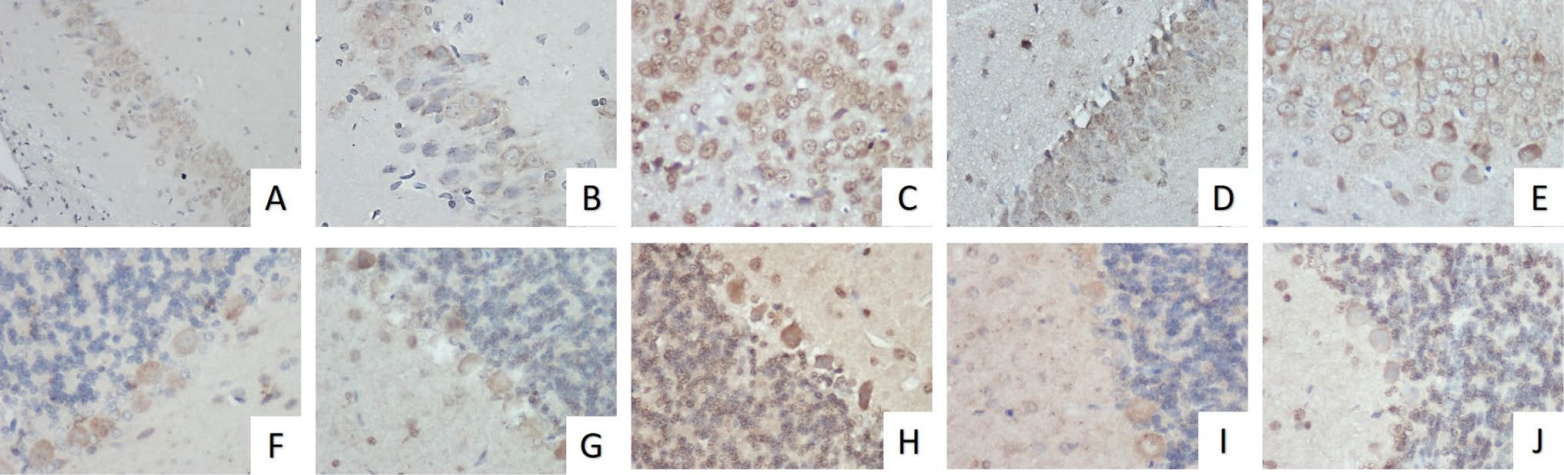
The intensity of Gal- 3 immunostaining in hippocampus and cerebellum tissue. **A**. Control group, hippocampus, X40. **B**. Sham group, hippocampus, X40. **C**. KA group, hippocampus, X40. **D**. TXR group, hippocampus, X40. **E**. KA + TXR group, hippocampus, X40. **F.** Control group, cerebellum, X40. **G**. Sham group, cerebellum, X40. **H.** KA group, cerebellum, X40. I. TXR group, cerebellum, X40. J. KA + TXR group, cerebellum, X40

In the KA group, neurons with dark eosinophilic cytoplasm and pyknotic nuclei were observed in the CA1 region of the hippocampus (Fig. 3C, D, I, J). In the cerebellum, a decrease in the number of Purkinje cells in the gangliosum layer was observed. These cells had pycnotic nuclei and dark eosinophilic cytoplasm, and showed reduced cell body size and irregular or spindle-shaped cell morphology (Fig. 4C, D, I, J). In the KA group, pyknotic nuclei in neurons and hemorrhagic areas in the granulosum layer were observed (Figs. 5C and D). These histopathological changes were reduced in the sections of the KA + TXR group compared to the KA group (Figs. 3F, L and 4F, L).

Histopathological evaluation of the hippocampus and cerebellum revealed significant neuronal damage in the KA group. As shown in Fig. 1, the number of pyknotic nuclei in the hippocampus was markedly increased in the KA group (2.46 ± 0.52) compared to the control (0.12 ± 0.33) and sham (0.14 ± 0.35) groups. The value in the TXR group (0.13 ± 0.34) was similar to the control and sham groups. With the application of KA + TXR (0.19 ± 0.39), the number of pyknotic nuclei observed in the KA group was significantly reduced (*P* < 0.001). Regarding the hemorrhagic areas in the hippocampus, the KA group showed a notable increase (0.45 ± 0.50) when compared to the control (0.01 ± 0.10), sham (0.06 ± 0.28), TXR (0.01 ± 0.10), and KA + TXR (0.07 ± 0.26) groups. No significant differences were observed among the control, sham, TXR, and KA + TXR groups. The administration of TXR in combination with KA significantly alleviated the hemorrhagic damage (*P* < 0.001). In the cerebellum, the number of pyknotic cells was significantly increased in the KA group (3.64 ± 0.98) compared to the control (1.11 ± 0.55) and sham (1.12 ± 0.54) groups. In contrast, the TXR (1.16 ± 0.60) and KA + TXR (1.19 ± 0.60) groups showed pyknotic cell counts similar to those of the control and sham groups. These findings indicate that TXR administration, particularly in combination with KA, mitigated the cerebellar neuronal damage caused by KA exposure (*P* < 0.001). As for the Purkinje cell count in the cerebellum, a significant reduction was observed in the KA group (4.89 ± 0.78) compared to the control (7.36 ± 0.75) and sham (7.37 ± 0.76) groups. The TXR (7.36 ± 0.75) and KA + TXR (7.34 ± 0.74) groups maintained Purkinje cell numbers close to the control group, indicating that TXR prevented Purkinje cell loss associated with KA-induced neurotoxicity (*P* < 0.001).

#### Immunohistochemical Findings

Immunohistochemical findings were evaluated according to Gal- 3 staining intensity. Gal- 3 expression was observed in the cell membrane and nucleus of neurons in the CA- 1 area of the hippocampus (Fig. 5A-E). H-Score of Gal- 3 expression was 147.17 ± 21.38 in the Control group, 150.67 ± 26.78 in the Sham group, 187.83 ± 18.72 in the KA group, 154.50 ± 7.34 in the TXR group, and 155.17 ± 6.46 in the KA + TXR group. While KA application caused a statistically significant increase in Galectin- 3 expression (*p* < 0.05), KA followed by TXR application caused a statistically significant decrease in Gal- 3 expression (*p* < 0.05). H-Score for Gal- 3 expression is shown in Table 2.

**Table 2:** Mean H-Score for Gal- 3 Immunoreactivity

Groups	H-score of hippocampus	H-score of cerebellum
Group 1: Control	147.17 ± 21.38	143.83 ± 19.33
Group 2: Sham	150.67 ± 26.78	140.33 ± 12.86
Group 3: KA	187.83 ± 18.72^a^	189.17 ± 18.38^b^
Group 4: TXR	154.50 ± 7.34	159.17 ± 7.96
Group 5: KA + TXR	155.17 ± 6.46	153.67 ± 2.88

Data are expressed as mean ± SD (*n* = 10). ^a^KA vs Control, Sham, TXR and KA + TXR *P* < 0.05. bKA vs Groups Control, Sham, TXR and KA + TXR *P* < 0.01

Gal- 3 expression was observed in the moleculare, gangliosum, and granulosum layers of the cerebellum (Fig. 5F-J). H-Score of Galectin- 3 expression was 143.83 ± 19.33 in the Control group, 140.33 ± 12.86 in the Sham group, 189.17 ± 18.38 in the KA group, 159.17 ± 7.96 in the TXR group, and 153.67 ± 2.88 in the KA + TXR group. While KA application caused a statistically significant increase in Galectin- 3 expression (*p* < 0.01), KA followed by TXR application caused a statistically significant decrease in Galectin- 3 expression (*p* < 0.01). H-Score for Gal- 3 expression is shown in Table 2.

## Discussion

Systemic administration of KA to rats stimulates glutamate release from presynaptic neurons, leading to glutamatergic excitotoxicity, a common pathological process in central nervous system diseases. It has been suggested that the appearance of oxidative stress partly causes this excitotoxicity [57] and leads to inflammatory responses and neuronal death in the cerebral cortex and hippocampus, causing progressive seizures [58]. Therefore, controlling glutamate release may be a helpful strategy for neuroprotection.

Systemic administration of KA to rats is an accepted model of temporal lobe epilepsy [13]. KA administration leads to spontaneous recurrent seizures and a continuous latent period leading to epilepsy [14]. Kainate receptors (KARs) modulate glutamate, and Gamma-Aminobutyric Acid (GABA) release [59], and the role of KARs in chronic temporal lobe epilepsy has recently been discovered [60, 61]. Alterations in the expression of KARs in the neural network can lead to long-lasting changes in glutamatergic connectivity, network excitability, and behaviourally relevant circuits in the brain [62]. AMPA receptors (AMPARs), one of the glutamate receptors, are the dominant mediators of glutamate-induced excitatory neurotransmission and determine synaptic efficiency and plasticity through their number and/or properties [1]. In experimental KA-induced epilepsy, hippocampal neuronal cell loss, as well as changes in proteins such as AMPARs, have been observed [63]. The presence of epilepsy involves changes in the trafficking, synaptic surface expression and signalling of kainate and AMPA receptors (KARs and AMPARs) [64]. KARs activation in hippocampal inhibitory interneurons has been shown to reduce GABA release. KAR activation-induced reduction in GABA release may increase seizure susceptibility [65]. KA produces epileptic effects that may be mediated by KAR activation (or AMPAR activation). In temporal lobe epilepsy, a severe and persistent disturbance, recurrent partial seizures originate from mesial structures such as the hippocampus [61]. All these data suggest that KA not only causes an increase in glutamate release [66], but may also have bidirectional effects on the modulation of excitatory transmission and affect GABA release through direct activation of KARs [1].

The pattern and extent of neuronal damage caused by systemic KA administration may depend on the spread of seizure activity to regions connected by excitatory neural pathways or on receptor subtype distribution [67]. It is suggested that the damage in hippocampal CA3 is related to kainate and NMDA receptors, and the damage in CA1 is related to AMPA and NMDA receptors [68]. It has been reported that overstimulation of CA3 by KA causes the stimulus to extend to CA1 via Schaffer collaterals immediately, and the released glutamate opens AMPA receptors [69]. Recent reports indicate that KA administration, which partially induces seizures through activation of AMPA receptors, suppresses calcium influx to presynaptic terminals and suppresses excitatory synaptic transmission at the CA1 region synapse of the hippocampus [70, 71]. In addition, blockade of AMPA receptors was reported to prevent CA1 hippocampal damage in adult rats [72]. Cell death caused by NMDA/AMPA/kainate receptor activation is attributed to ROS generation. In the absence of oxygen, cells can increase intracellular calcium levels. This suggests that ROS may be an important effector of toxicity elicited by increased intracellular calcium [73, 74].

In this study, we investigated for the first time the effect of TXR treatment on KA-induced excitotoxicity and its relationship with Gal- 3 expression. The most important finding in our study is that TXR treatment has a neuroprotective effect in the KA-induced excitotoxicity model by providing significant improvements in cerebral cortex MDA and GSH levels, decreasing cerebral cortex IL- 1β levels and decreasing Gal- 3 expression in the CA1 region of the hippocampus and various layers of the cerebellum.

The increase in reactive oxygen species (ROS) and inadequate antioxidant defences render the brain vulnerable to oxidative damage, leading to the development of many inflammatory and degenerative neurological diseases [75, 76]. Although oxidative stress and neuroinflammation seem to be two different pathological events [77], inflammation triggered by oxidative stress is the cause of many chronic diseases [78]. GSH acts as an important ROS scavenger by eliminating the dangerous effects of reactive oxidant molecules [79–81]. Another sensitive biomarker of oxidative stress is the lipid peroxidation end product MDA [82]. A study investigating the effect of salidroside on KA-induced status epilepticus in mice showed that MDA levels increased and GSH production decreased in KA-induced pathology [83]. In a study investigating the effects of nicotinamide adenine dinucleotide phosphate (NADPH) and Mito-apocynin, a NOX inhibitor, in a neuronal excitotoxicity model induced by stereotypically injecting KA into the unilateral striatum of mice, it was found that KA caused an increase in MDA concentration [84]. In another study in which KA-induced neurotoxicity and oxidative stress model was established and the functional role of Human programmed cell death 4 (PDCD4) was investigated in HT22 cells, it was observed that KA administration significantly increased MDA levels compared to the control group [85]. However, another study suggests that brain regions such as hypothalamus, striatum and cerebral cortex are less sensitive to KA excitotoxicity and that the pattern of oxidative damage caused by systemic KA administration is region-specific [86]. In a study evaluating the neuroprotective potential of cynomenine as well as its effect on oxidative stress and inflammation in a rat model of temporal lobe epilepsy induced by intrahippocampal kainate, it was reported that GSH production decreased, ROS and MDA levels increased and Nuclear Factor kappa B (NFκB) and Tumour necrosis factor-alpha (TNF-α) levels increased [87].

It has been established that IL- 1β plays a pivotal role in instigating the initial phases of inflammation. Moreover, IL- 1β has been identified as an important factor in cases of neuroinflammation associated with certain forms of neurodegeneration, including trauma and excitotoxic brain injury [88]. In a study examining the relationship between convulsants and IL- 1β mRNA in the rat brain, it was found that IL- 1β mRNA was intensely induced in the cerebral cortex, thalamus and hypothalamus, moderately induced in the hippocampus but not in the midbrain and cerebellum after convulsive dose (10 mg/kg-ip) of KA administration [89]. In a study investigating the effect of IRFI 042, a lipid peroxidation inhibitor, on brain damage induced by KA administration in rats, it was reported that MDA increased significantly in the cortex and hippocampus and GSH production decreased. In addition, it was shown that IL- 1β levels in cerebral cortex tissue increased after KA administration. [90]. In cyclophosphamide-induced 10-day brain damage in rats, a significant increase in MDA (91%), a decrease in GSH brain content (22%) and a 64% increase in IL- 1β brain content were reported compared to the normal control group [91]. In a study with epileptic rats induced by KA injection into the right CA3 region of the hippocampus, KA administration caused a significant increase in IL- 1β content, as well as a significant increase in MDA concentration and a decrease in GSH level [92]. All these studies show that oxidative damage and lipid peroxidation increase and antioxidant defence mechanisms collapse in KA-induced excitotoxicity. The present study revealed that excitotoxicity induced by KA administration resulted in elevated MDA levels and diminished GSH synthesis in the model group.

The results of the present study suggest that KA administration may stimulate glutamate receptors and increase glutaminergic activity by increasing ROS levels. In our study, increased oxidative stress as a result of icv KA administration suppressed GSH levels and caused a significant decrease in GSH levels. This indicates that GSH is consumed to overcome oxidative stress by neutralising these radicals as a result of a reaction with superoxide radicals. In addition, increased ROS and lipid peroxidation induced an increase in MDA levels.

TXR exhibits numerous favourable pharmacological properties, including antioxidants, anti-inflammatory, and antiapoptotic properties [93, 94]. In many studies, it has been shown that it has neuroprotective effects by reducing oxidative stress and improving angiogenesis [95], renoprotective effects by reducing inflammation and kidney damage [96], and hepatoprotective [97] and cardioprotective effects by reducing oxidative stress and inflammation [98]. In a study investigating the potential neuroprotection mechanisms of TXR against lipopolysaccharide (LPS)-induced oxidative stress and neuroinflammation, it was determined that ROS production, NFκB, TNF-α and MDA levels increased in LPS model group rats. It was then reported that TXR treatment significantly suppressed LPS-induced acute neuroinflammation, oxidative stress and apoptosis [42]. In another study investigating the effect of TXR on ischaemic/reperfusion-induced brain injury and neuroinflammatory oxidative stress in brain tissues in rats, it was found that TXR (10 ve 20 mg/kg/bw) administration significantly increased SOD, catalase and GSH while restoring MDA and acetylcholine esterase levels and improving behavioural and neural activity [99]. In the intrastriatal 6-hydroxydopamine-induced Parkinson's disease model in rats, TXR treatment at a dose of 150 mg/kg/day for 1 week decreased striatal MDA and ROS levels as lipid peroxidation index and DNA fragmentation as an apoptotic marker [100]. In another study investigating the relationship between oxidative stress and neuroinflammatory response in an LPS-induced depression model in mice and TXR treatment, it was revealed that TXR increased GSH and SOD levels and decreased MDA, TNF-α, Interleukin 6 and interferon-gamma levels compared to negative control [101]. Our study observed decreased cerebral cortex tissue MDA levels and a significant increase in GSH levels with TXR treatment. In addition, TXR treatment showed a suppressive effect on neuroinflammation by decreasing IL- 1β levels.

Recently, Gal- 3 has attracted attention as an important component in the mechanisms underlying oxidative stress-induced inflammatory reaction and fibrogenesis [102]. Many studies have reported that Gal- 3 levels increase in the pathogenesis of neurodegenerative diseases [103–105]. A study reported that genetic down-regulation or pharmacological inhibition of Gal- 3 may represent a new therapeutic target in Parkinson's disease and other synucleinopathies. This study suggests that inhibition of the activity of this protein leads to a significant reduction in the inflammatory response [32]. In another study investigating the role of Gal- 3, a microglial marker, in the neurodegenerative mechanism of frontotemporal dementia (FTD), high levels of Gal- 3 were found in the cerebrospinal fluid and serum of both sporadic and genetic FTD patients [106]. Gal- 3 has also been shown to be upregulated in the brains of Alzheimer's patients and 5xFAD (familial Alzheimer's disease) mice and is expressed explicitly in microglia associated with amyloid beta (Aβ) plaques [104, 107]. Furthermore, Gal- 3 is considered a potential oxidative stress biomarker in forming reactive oxygen species and GSH depletion [108–110]. Significantly higher levels of Gal- 3 have been reported in neocortical and hippocampal tissues of patients with early-onset Alzheimer's disease, including genetic and sporadic cases. [111]. An experimental intracerebral haemorrhage model study found that increased the Gal- 3 expression level of rats exacerbated brain damage due to intracerebral haemorrhage by increasing neuroinflammatory activation and nerve cell death. [112]. A study showed that the increase in Gal- 3 levels in Huntington's disease patients and the brain tissues of mice with Huntington's disease was associated with the severity of the disease. It has also been observed that Gal- 3 up-regulation in mice remains elevated in microglia throughout disease progression and promotes inflammation [105]. Another study reported direct correlations between plasma Gal- 3 levels and oxidative stress and inflammation markers. [113]. In a study using the controlled cortical impact model of head injury and investigating Gal- 3 levels in microglia and Gal- 3 levels released from cerebrospinal fluid, a significant increase in Gal- 3 expression was found. However, Gal- 3 neutralising antibody treatment has been reported to reduce trauma-induced increases in pro-inflammatory markers such as IL- 1β, IL- 6 and TNF-α, supporting neuroprotection in cortical and hippocampal cell populations after head injury. This neuroprotection was also observed in Gal- 3 knockout animals [114].

Our study found that Gal-3 expression in the cell membrane and nucleus of neurons in the CA1 region of the hippocampus was higher in KA-treated rats than in the control and sham groups. However, a decrease in Gal- 3 expression was detected in the KA + TXR group that received TXR treatment compared to the KA group. Gal- 3 expression increased in the molecular, gangliosum and granular layers of the cerebellum as a result of KA administration. In contrast, the KA + TXR group treated with TXR showed a strong decrease in Gal- 3 expression compared to the KA group. These data suggest that increased Gal- 3 expression is harmful and may trigger the development of neurodegeneration.

It has been reported that injection of KA into the cerebellum leads to a rapid destruction of stellate, basket and Golgi II cells, especially Purkinje cells [22], and a neuroexcitatory and neurotoxic effect on neurons associated with cerebellar granule cells [115]. A study found that administering low doses of cerebellar KA to rats caused rapid and selective destruction of neurons, depletion of Purkinje cells in a very short time following the injection, and a significant depletion of granule cells in about a week [116]. In short-term experiments, it was observed that KA did not affect granule cells in monolayer cultures of fetal rat cerebellum but showed cytotoxic effects on Purkinje neurons [117].

Uninjured mature neurons undergo apoptosis in response to an exogenous toxic stimulus. Pyknotic nuclei observed in dying neurons indicate cell death occurs through apoptosis [118]. In addition, preventing the appearance of pyknotic nuclei from pretreatment with a therapeutic agent confirms the neuroprotective effect. [119]. In our study, neurons with dark eosinophilic cytoplasm and pyknotic nuclei were observed in the CA1 region of the hippocampus of KA-treated rats, and a decrease in the number of Purkinje cells in the gangliosum layer was observed in the cerebellum of KA-treated rats. These cells have pyknotic nuclei and dark eosinophilic cytoplasm, suggesting cell death by apoptosis. However, these histopathological changes decreased in the KA + TXR group treated with TXR treatment compared to the KA group. In this study, we focused on the qualitative assessment of cellular damage markers, providing valuable insights into their characteristics. Quantitative methods such as stereological analysis could have further increased the depth of our findings. Future research integrating these methodologies could build upon our results, offering a more comprehensive understanding of cellular damage. Recognizing this as an opportunity for further exploration, we encourage future studies to incorporate quantitative approaches to strengthen and expand upon our observations.

The findings of this study demonstrated that treatment with TXR can attenuate KA-induced neurotoxicity by reducing oxidative tissue damage, inflammatory response and Gal- 3 expression. These data also provide experimental evidence supporting that Gal- 3 correlates with increased oxidative stress and neuroinflammatory activation. Gal- 3 may be a target for developing therapeutic agents in pathologies associated with neurotoxicity, while TXR may have therapeutic potential in many diseases related to oxidative stress and inflammation, including neurotoxicity.

## Data Availability

No datasets were generated or analysed during the current study.
